# Supplementary material for: Heat adaptation of phage T7 under an extended genetic code
Source: Virus Evol. 2021 Dec 1;7(2):veab100. doi: 10.1093/ve/veab100 (PMC8923235; doi:10.1093/ve/veab100)
Supplement: veab100_Supp [file veab100_supp.zip › Sequencing_Analysis.zip!/Sequencing_Analysis/A1/evidence/SNP_8.html]

BRESEQ :: Evidence


| Predicted mutation | | | | | | |
| --- | --- | --- | --- | --- | --- | --- |
| evidence | seq id | position | mutation | annotation | gene | description |
| RA | NC\_001604.1 | 27,783 | G→A | intergenic (–/–) | – / – | –/– |

| Read alignment evidence... | | | | | | | | | | | |
| --- | --- | --- | --- | --- | --- | --- | --- | --- | --- | --- | --- |
|  | seq id | position | | ref | new | freq | score (cons/poly) | reads | annotation | genes | product |
| \* | NC\_001604.1 | 27,783 | 0 | G | A | 96.0% | 6853.7 / 149.2 | 3183 | intergenic (–/–) | *–/–* | –/– |
| Reads supporting (aligned to +/- strand):  **ref** base G (63/63);  **new** base A (1746/1275);  **total** (1830/1353) | | | | | | | | | | | |
| Fisher's exact test for biased strand distribution *p*-value = 9.75e-02 | | | | | | | | | | | |
| Kolmogorov-Smirnov test that lower quality scores support variant *p*-value = 1.00e+00 | | | | | | | | | | | |

Only 100 of 3190 total aligned reads displayed.  

`GTGCTGGTTCGTTACGAGCGACCAAGTGTGGCGACTTAGTGGAAAGGCTAAGCGAAAGTTCCGTAAGTTAATCATGGAGTATCGCGATAAGATGCTTGAGAAGTATGATACTCTTTGGAATTACGTATGGGTAGGCAATACGTCCCACATTCGTTTCCTCAAGACTATCGGTGCGGTATTCCATGAAGAGTACACACGAGATGGTCAATTTCAGTTATTTACAATCACGAAAGGAGGATAACCATATGTGTTGGGCAGCCGCAATACCTAT‑‑‑‑‑‑‑‑‑‑‑‑CGCTATATCTGGCGCTCAGGCTATCAGTGGTCAGAACGCTCAGGCCAAAATGATTGCCGCTCAGACCGCTGCTGGTCGTCGTCAAGCTATGGAAATCATGAGGCAGACGAACATCCAGAATGCTGACCTATCGTTGCAAGCTCGAAGTAAACTTGAGGAAGCGTCCGCCGAGTTGACCTCACAGAACATGCAGAAGGTCCAAGCTATTGGGTCTATCCGAGCGGCTATCGGAGAGAGTATGCTTGAAGGTTCCTCAATGGACCGCATTAAGCGAGTCACAGAAGGACAGTTCATTCGGGAAGCCAATATGGTAACTGAGAACTATCGCCG  >  NC_001604.1/27483‑28083`  

                                                                                                                                                                                                                                                                                                                        `|`                                                                                                                                                                                                                                                                                                              
     `ggTTCGTTACGAGCGACCAAGTGTGGCGAATTAGTGGAAAGGCTAAGCGAAAGTTCCGTAAGTTAATCATGGAGTATCGCGATAAGATGCTTGGGAAGTATGATACTCTTTGGAATTACGTATGGGTAGGCAATACGTCCCACATTCGGTTCCTCAAGACTATCGATGCGGTATTCCATGAAGAGTACACACGAGATGGTCAATTTCAGTTATTTACAATCACGAAAGGAGGATAACCATATGTGTTGGGCAGCCGCAATACCTAT‑‑‑‑‑‑‑‑‑‑‑‑CGCTATATCTGGCGATCAGGCTATCAGTGATCAGa                                                                                                                                                                                                                                                                                                         <  1:30762/301‑1 (MQ=255)`  
                      `cAAGGGTGGCGACTTAGTGGAAAGGCTAAGCGAAAGTTCCGTAAGTTAATCATGGGGTATCGCGATAAGATGCTTGAGAAGTATGATACTCTTTGGAATTACGTATGGGTAGGCAATACGTCCCACATTCGTTTCCTCAAGACTATCGGTGCGGTATTCCATGAAGAGTACACACGAGATGGTCAATTTCAGTTATTTACAATCACGAAAGGAGGATAACCATATGTGTTGGGCAGCCGCAATACCTAT‑‑‑‑‑‑‑‑‑‑‑‑CGCTATATCTGGCGCTCAGGCTATTAGTGATCAGAACGCTCAGGCCAAAATg                                                                                                                                                                                                                                                                                        <  1:184443/301‑1 (MQ=255)`  
                         `gtgtGGCGACTTAGTGGAAAGGCTAAGCGAAAGTTCCGTAAGTTAATCATGGAGTATCGCGATAAGATGCTTGAGAAGTATGATACTCTTTGGAATTACGTATGGGTAGGCAATACGTCCCACATTCGTTTCCTCAAGACTATCGGTGCGGTATTCCATGAAGAGTACACACGAGCTGGCCAATTTCAGTTATTTACAATCACGAAAGGAGGATAACCATATGTGTTGGGCAGCCGCAATACCTAT‑‑‑‑‑‑‑‑‑‑‑‑CGCTATATCTGGCGCTCAGGCTATCAGTGATCAGAACGCTCACGcctgtctctta                                                                                                                                                                                                                                                                                     >  1:404282/1‑292 (MQ=255)`  
                         `gtgtGGCGACTTAGTGGAAAGGCTAAGCGAAAGTTCCGTAAGTTAATCATGGAGTATCGCGATAAGATGCTTGAGAAGTATGATACTCTTTGGAATTACGTATGGGTAGGCAATACGTCCCACATTCGTTTACTCAAGACTATCGGTGCGGTATTCCATGAAGAGTACACACGAGATGGACAATTTAAGTTATTTAAAATCACGAACGGAGGATAACCATATGTGTTGGGCAGCCGCAATACCTAT‑‑‑‑‑‑‑‑‑‑‑‑CGCTATATCTAGCGCTCAGTCTATGAGAGATCATAACCCTCAGGCCAAAATGAtt                                                                                                                                                                                                                                                                                     >  1:48152/1‑301 (MQ=255)`  
                                `agacaggTGGAAAGGCTAAGCGAAAGTTCCGTAATTTAATCCTTGATTTTCGCGATAAGATGCTTGAGAAGTATGATACTCTTTGGAATTACGTATGGGTAGTCAATACGTCCCACATTCGTTTCCTCAAGACTATCGGTGCGGTATTCCATGAAGATTACACACGAGATGGTCAATTTCAGTTATTTACAATCACGAAAGGAGGATAACCATATGTGTTGGGCAGCCGCAATACCTAT‑‑‑‑‑‑‑‑‑‑‑‑CGCTATATCTGGCGCTCAGGCTATCAGTGATCAGAACGCTCAGGCCAAAATGATTGCAGCTc                                                                                                                                                                                                                                                                              <  1:467003/295‑1 (MQ=255)`  
                                  `cTTAGTGGAAAGGCTAAGCGAAAGTTCCGTAAGTTAATCATGGAGTATCGCGATAAGATGCTTGAGAAGTATGATACTCTTTGGAATTACGTATGGGTAGGCAATACGTCCCACATTCGTTTCCTCAAGACTATCGGTGCGGTATTCCATGAAGAGTACACACGAGATGGTCAATTTCAGTTATTTACAATCACGAAAGGAGGATAACCATATGTGTTGGGCAGCCGCAATACCTAT‑‑‑‑‑‑‑‑‑‑‑‑CGCTATATCTGGCGCTAAGGCTATCAGTGATCAGAACGCTCAGGCCAAAATGATTGCCGCTCaa                                                                                                                                                                                                                                                                            >  1:413845/1‑300 (MQ=255)`  
                                    `tAGTGGAAAGGCTAAGCGAAAGTTCCGTAAGTTAATCATGGAGTATCGCGATAAGATGCTTGAGAAGTATGATACTCTTTGGAATTACGTATGGGTAGGCAATACGTCCCACATTCGTTTCCTCAAGACTATCGGTGCGGTATTACATGAAGAGTACACACGAGATGGTCAATTTAAGTTATTTAAAATCACGAAAGGAGGATAACCATATGTGTTGGGCAGCCGAAATACATCT‑‑‑‑‑‑‑‑‑‑‑‑CGCTATATCTGGCGCTCAGGCTATCATTGATCAGAACGCTCAGGCCAACATGATAGCCGCTCAGaa                                                                                                                                                                                                                                                                          >  1:143958/1‑300 (MQ=255)`  
                                              `gCTAAGCGAAAGTTCCGTAAGTTAATCATGGAGTATCGCGATAAGATGCTTGAGAAGTATGATACTCTTTGGAATTACGTATGGGTAGGCAATACGTCCCACATTCGTTTCCTCAAGACTATCGGTGCGGTATTCCATGAAGAGTACACACGAGATGGTCAATTTCAGTTATTTACAATCACGAAAGGAGGATAACCATATGTGTTGGGCAGCCGCAATACCTAT‑‑‑‑‑‑‑‑‑‑‑‑CGCTATATCTGGCTCTCAGGCTATCAGTGATCAGAACGCTCAGGCCAAAATGATTGCCGCTCAGACCGCTGCTGgt                                                                                                                                                                                                                                                                <  1:3505/301‑1 (MQ=255)`  
                                               `ggagatgtgtattatctacaatAATCATGGTGTATCACGATAAGATGCTTGAGAAGGATGATACTATTTGGAATTACGTATGGTTAGGCAATACGTCCCACATTCGTTTCCTCAAGACTATCGGTGCGGTATTCCATGAAGAGTACACACGAGATGGTCAATTTCAGTTATTTACAATCACGAAAGGAGGATAACCATATGTGTTGGGCAGCCGCAATACCTAT‑‑‑‑‑‑‑‑‑‑‑‑CGCTATATCTGGCGCTCAGGCTATCAGTGATCAGAACGCTCAGGCCAAAATGATTGCCGCTCAGACCGCTGCTGgtc                                                                                                                                                                                                                                                               <  1:109065/280‑1 (MQ=255)`  
                                                         `gTTCAGTAAGTGAAGCAGGGAGTATAGGGATAAGATGCTTGAGAAGTATGATACTATTTGGAATGACGTATGGTGAGGAAATACGTCCCACATTCGTTTCCTCAAGACTATCGGTGCGGTATTCCATGAAGAGTACACACGAGATGGTCAATTTCAGTTATTTACAATCACGAAAGGAGGATAACCATATGTGTTGGGCAGCCGCAATACCTCT‑‑‑‑‑‑‑‑‑‑‑‑CGCTATATCTGGCGCTCAGGCTATCAGTGATCAGAACGCTCAGGCCAAAATGATTGCCGCTCAGACCGCTGCTGGTCGTCGTCAAGc                                                                                                                                                                                                                                                     <  1:39331/301‑1 (MQ=255)`  
                                                          `ttCCGTAAGTTAATCATGGAGTATCGCGATAAGATGCTTGATAAGTATGATACTCTTTGGAATTACTTATGGGTAGGCAATACGTCCCACATTCGTTTCCTCAAGACTATCGGTGCGGTATTCCATGAAGAGTACACACGAGATTGTCAATTTCAGTTATTTACAATCACGAAAGGAGGATAACCATATGTGTTGGGCAGCCGCAATACCTAT‑‑‑‑‑‑‑‑‑‑‑‑CGCTATATCTGGCGCTCAGGCTATCAGTGATCAGAACGCTCAGTCCAAAATGATTGCCGCTCAGACCGCTGCTGGTCGTCGTCAAGCt                                                                                                                                                                                                                                                    <  1:100077/301‑1 (MQ=255)`  
                                                                      `aTCATGGAGTATCGCGATAAGATGCTTGAGAAGTATTATACTCTTTGGAATTACGTATGGGTAGGCAATACGTCCCACATTCGTTTCCTCAAGACTATCGGTGCGGTATTCCATGAAGAGTACACACGAGATGGTCACTTTCAGTTATTTACAATCACGAAAGGAGGATAACCATATGTGTTGGGCAGCCGCAATACCTAT‑‑‑‑‑‑‑‑‑‑‑‑CGCTATATCTGGCGCTCAGGCTATCAGTGATCAGAACGCTCAGGCCAAAATGATTGCCGCTCAGACCGCTGCTGGTCGTCGTCAAGcctgtctcttatac                                                                                                                                                                                                                                        >  1:228632/1‑288 (MQ=255)`  
                                                                            `gAGTATCGCTATAAGATGCTTGAGAAGTATGATACTCTTTGGAATTACGTATGGGTAGGCAATACGTCCCACATTCGTTTCCTCAAGACTATCGGTGCGGTATTCCATGAAGAGTACACACGAGATGGTCAATTTCAGTTATTTACAATCACGAAAGGAGGATAACCATATGTGTTGGGCAGTCGCAATACCTAT‑‑‑‑‑‑‑‑‑‑‑‑CGCTATATCTGGCGCTCAGGCTATCAGTGATCAGAACGCTCAGGCCAAAATGATTGCCACTCAGACCGCTGCTGGTCATCGTCACGCTATGGAAATCATTATGCAg                                                                                                                                                                                                                                  >  1:48758/1‑301 (MQ=255)`  
                                                                            `gAGTATCGCGATAAGATGCTTGAGAAGTATGATACTCTTTGGAATTACGTATAGGTAGGCACTACGTCCCACATTCGTTTCCTCAATACTATCGGTGCGGTATTCCATGAAGAGTACACACGAGATGGACAATTTCCATTTTTTACACTCCCGACAGGAGGATAACCATATTTTTTCGGCAGCCGCACTACCTAT‑‑‑‑‑‑‑‑‑‑‑‑CGCTATATCTGACACTCAGACTATCAGTGATCAGAACGCCAACGCCAAAATGCTTGCCGCTCAGACCGCGTATTGTACTCGTCACTCTATGATACTCATGAGCCac                                                                                                                                                                                                                                  >  1:504802/1‑300 (MQ=255)`  
                                                                                `aTCGCGATAAGATGCTTGAGAAGTATGATACTCTTTGGAATTACGTATGGGTAGGCAATACGTCCCACATTCGTTTCCTCAAGACTATCGGTGCGGTATTCCATGAAGAGTACACACGAGATGGTCAATTTCAGTTATTTACAATCACGAAAGGAGGATAACCATATGTGTTGGGCAGCCGCAATACCTAT‑‑‑‑‑‑‑‑‑‑‑‑CGCTATATCTGGCGCTCAGGCTATCAGTGATCAGAACGCTCAGGCCAAAATGATTGCCGCTCAGACCGCTGCTGGTCGTCGTCAAGCTATGGAAATCATGAGGCAGACGa                                                                                                                                                                                                                              <  1:149053/301‑1 (MQ=255)`  
                                                                                      `aTAAGATGCTTGAGAAGTATGATACTCTTTGGAATTACGTATGGGTAGGCAATACGTCCCACATTCGTTTCCTCAAGACTATCGGTGCGGTATTCCTTGAAGAGTACACACGAGATGGTCAATTTCAGTTATTTACAATCACGAAAGGAGGATAACCATATGTGTTGGGCTGCCGCAATACCTAT‑‑‑‑‑‑‑‑‑‑‑‑CGCTATATCTGGCGCTCAGGCTATCAGTGATCAGAACGCTCAAGCCAAAATGATTGCCGCTCAGACCGCTGCTGGTCGTCGTCAAGCTATGGAAATCATGAGGCAGACGACCATcc                                                                                                                                                                                                                        >  1:215512/1‑301 (MQ=255)`  
                                                                                             `gCTTGAGACGTATGATACTCTTTGGAATTACGTATGGGTAGGCAATACGTCACACATTCGTTTCCTCAAGACTATCGGTGCGGTATTCCATGAAGAGTACACACGAGATGGTCAATTTCAGTTATTTACAATCACGAAAGGAGGATAACCATATGTGTTGGGCAGCCGCAATACCTAT‑‑‑‑‑‑‑‑‑‑‑‑CGCTATATCTGGCGCTCAGGCTATCAGTGATCAGAACGCTCAGGCCAAAATGATTGCCGCTAAGACCGCTGCTGGTCGTCGTCAAGCTATGGAAATCATGAGGCAGACGAACATCCAGAATGc                                                                                                                                                                                                                 >  1:472702/1‑301 (MQ=255)`  
                                                                                             `gCTTGAGAAGTATGATACTCTTTGGAATTACGTATGGGTAGGCAATACGTCCCACATTCGTTTCCTCAAGACTATCGGTGCGGTATTCCATGAAGAGTACACACGAGATGGTCAATTTCAGTTATTTACAATCACGAAAGGAGGATAACCATATGTGTTGGGCAGCCGCAATACCTAT‑‑‑‑‑‑‑‑‑‑‑‑CGCTATATCTGGCGCTCAGGCTATCAGTGATCAGAACGCTCAGGCCAAAATGATTGCCGCTCAGACCGCTGCTGGTCGTCGTCAAGCTATGGAAATCATGAGGCAGACGAACATCCAGAATGc                                                                                                                                                                                                                 <  1:202575/301‑1 (MQ=255)`  
                                                                                             `cgttGAAAAGTATGTTTATTATTTGAATTAAATATGTGTAGGGAATACGTCACACATTCGTTTCCACAAGCCTAACGGTGGGTTATTCCATGAAGAATAGAAACCAGATGGTCAATTTCAATTATTTACAATCACGAAAGGAGGATAACCATATGTGTTGGGCAGCCGCAATACCTAT‑‑‑‑‑‑‑‑‑‑‑‑CGCTATATCTGGCGCTCAGGCTATCAGTGATCAGAACGCTCAGGCCAAAATGATTGCCGCTCAGACCGCTGCTGGTCGTCGTCAAGCTATGGAAATCATGAGGCAGACAAACATCCAGAATGc                                                                                                                                                                                                                 <  1:119601/299‑1 (MQ=255)`  
                                                                                              `cTTGAGAAGTATGATACTCTTTGGAATTACGTATGGGTAGGCAATACGTCCCACATTCGTTTCCTCTAGACTATCGGTGCGGTATTCCATGAAGAGTACACACGAGATGGTCAATTTCAGTTATTTACAATCACGAAGGGAGGATAACCATATGTGTTGGGCAGCCGCAATACCTAT‑‑‑‑‑‑‑‑‑‑‑‑CGCTATATCTGGCGCTCAGGCTATCAGTGATCAGAACGCTCAGGCCAAAATGATTGCCGCTCAGACCGCTGCTGGTCGTCGTCAAGCTATGGAAATCATGAGGCAGACGAACATCCAGAATGCt                                                                                                                                                                                                                >  1:237116/1‑301 (MQ=255)`  
                                                                                                    `aaGTATGATACTCTTTGGAATTACGTATGGGTAGGCAATACGTCCCACATTCGTTTCCTCAAGACTATCGGTGCGGTATTCAATGGAGAGTACACACGAGATGGTCAATTTCAGTAATTTACAATCACGAAAGGAGGATAACCATATGTGTATGGCAGCCGCAATACCTAT‑‑‑‑‑‑‑‑‑‑‑‑CGCTATATCTGGCGCTCAGGCTATAAGTGATCAGAACGCTAAGGCCACAATGATTGCCGCTCAGACCGATGCTGGTCGTCGTGAAGCTATGGAAATCATGAGGCAGACGAACATCAAGAATGCTGCCCTa                                                                                                                                                                                                          >  1:450466/1‑301 (MQ=255)`  
                                                                                                     `aGTATGATACTCTTTGGAATTACGTATGGGTAGGCAATACGTCCCACATTCGTTTCCTCAAGACTATCGGTGCGGTATTCCATGAAGAGTACACACGAGATGGTCAATTTCAGTTATTTACAATCACGAAAGGAGGATAACCATATGTGTTGGGCAGCCGCAATACCTAT‑‑‑‑‑‑‑‑‑‑‑‑CGCTATATCTGGCGCTCAGGCTATCAGTGATCAGAACGCTCAGGCCAAAATGATTGCCGCTCAGACCGCTGCTGGTCGTCGTCAAGCTATGGAAATCATGAGGCAGACGAACATCCAGAATGCTGACCTAt                                                                                                                                                                                                         <  1:458787/301‑1 (MQ=255)`  
                                                                                                      `gTATGATACTCTTTGGAATTACGTATGGGTAGGCAATACGTCCCACATTCGTTTCCTCAAGACTATCGGTGCGGTATTCCATGAAGAGTACACACGAGATGGTCAATTTCAGTTATTTACAATCACGAAAGGAGGATAACCATATGTGTTGGGCAGCCGCAATACCTAT‑‑‑‑‑‑‑‑‑‑‑‑CGCTATATCTGGCGCTCAGGCTATCAGTGATCAGAACGCTCAGGCCAAAATGATTGCCGCTCAGACCGCTGCTGGTCGTCGTCAACCTATGGAAATAATGAGGCAGACGAACATCCAGAATGCTTACCTATc                                                                                                                                                                                                        >  1:333364/1‑301 (MQ=255)`  
                                                                                                              `ctctTTGGCATTACGTATGGGTAGGCAATACGTCCCACATTCGTTTCCTCAAGACTATCGGTGCGGTATTCCATGAAGAGTACACACGAGATGGTCAATTTCAGTTATTTACAATCACGAAAGGAGGATAACCATATTTGTTGGGCAGCCGCCATACCTAT‑‑‑‑‑‑‑‑‑‑‑‑CGCTATATCTGGCGCTCAGGCTATCAGTGATCATAACGCTCAGGCCAAAATTATTGCCGCTCAGACCGCTGCTGGTCATCGTCCAGCTCTGAAACTCCTTAGGCATACGAACCTCCAGAATCCTGACCTATCGTTGCaat                                                                                                                                                                                                >  1:32556/1‑300 (MQ=255)`  
                                                                                                                `ctTTTGAATTACGTATGGGTAGGCAATACGTCCCACATTCGTTTCCTCAAGACTATCGGTGCGGTATTCCATGAAGAGTACACACGAGATGGTCAATTTCAGTTATTTACAATCACGAAAGGAGGATAACCATATGTGTTGGGCAGCCGCAATACCTAT‑‑‑‑‑‑‑‑‑‑‑‑CGCTATATCTGGCGCTCAGGCTATCAGTGATCAGAACGCTCAGGCCAAAATGATTGCCGCTCAGACCGCTGCTGGTCGTCGTCAAGCTATGGAAATCATTAGGCAGACGCACATCCAGAATGCTGACCTATCGTTGCCAGCt                                                                                                                                                                                              >  1:34552/1‑301 (MQ=255)`  
                                                                                                                     `gAATTACGTATGGGTAGGCAATACGTCCCACATTCGTTTCCTCAAGACTATCGGTGCGGTATTCCATGAAGAGTACACACGAGATGGTCAATTTCAGTTATTTACAATCACGAAATGAGGATAACCATATGTGTTGGGCAGCCGCAATACCTAT‑‑‑‑‑‑‑‑‑‑‑‑CGCTATATCTGGCGCTCAGGCTATCAGTGATCAGAACGCTCAGGCCAAAATGATTGCCGCTCAGACCGCTGCTTGTCGTCGTCAAGCTATGGAAATCATGAGGCAGACGAACATCCAGAATGCTGACCTATCGTTGCAAGCTCGAAg                                                                                                                                                                                         >  1:440973/1‑301 (MQ=255)`  
                                                                                                                          `aCGTATGGGTAGGCAATACGTCCCACATTCGTTTCCTCAAGACTATCGGTGCGGTATTCCATGAAGAGTACACACGAGATGGTCAATTTCAGTTATTTACAATCACGAAAGGAGGATAACCATATGTGTTGGTCAGCCGCAATACCTAT‑‑‑‑‑‑‑‑‑‑‑‑CGCTATATCTGGCGCTCAGGCTATCAGTGATCAGAACGCTCAGGCCAAAATGATTGCCGCTCAGACCGCTGCTGGTCGTCGTCAAGCTATGGAAATCATGAGGCAGACGAACATCCAGAATGCTGACCTATCGTTGCAAGCTCGAAgctgtc                                                                                                                                                                                    >  1:455031/1‑296 (MQ=255)`  
                                                                                                                          `aCGTATGGGTAGGCAATACGTCCCACAATCGGTTCCTCAAGACTATCGGGGCGGTATTCCATGAAGAGTACACACGAGATGGTCAATTTCAGTTATTTACAATCACGAAAGGAGTAGAACCATATGTGTTGTGCAGCCGCAATACCTAT‑‑‑‑‑‑‑‑‑‑‑‑CGCTATATCTGGCGCTCAGGCTATCAGTGATCAGAACGCTCAGGCCAAAATGATTGCCGCTCAGACCGCTGCTGGTCGTCGTCAAGCTATGGAAATCATGAGGCAGACGAACATCCAGAATGCTGACCTATCGTTGCAAGCTCGAAGTAAAc                                                                                                                                                                                    <  1:501334/301‑1 (MQ=255)`  
                                                                                                                                 `ggTAGGCAATACGTCCCACATTCGTTTCCTCAAGACTATCGGTGCGGTATTCCATGAAGAGTACACACGAGATGGTCAATTTCAGTTATTTACAATCACGAAAGGAGGATAACCATATGTGTTGGGCAGCCGCAATACCTAT‑‑‑‑‑‑‑‑‑‑‑‑CGCTATATCTGGCGCTCAGGCTATCAGTGATCAGAACGCTCAGGCCAAAATGATTGCCGCTCAGACCGCTGCTGGTCGTCGTCAAGCTATGGAAATCATGAGGCAGACGAACATCCCGCATGCTGACCTATCGTTGCAAGCTCGAATTAAACTTGAGGa                                                                                                                                                                             >  1:105628/1‑301 (MQ=255)`  
                                                                                                                                    `agacAATACGTCCCACATTCGTTTCCTCAAGACTATCGGTGCGGTATTCCATGAAGAGTACACACGAGATGGTCAATTTCAGTTATTTACAATCACGAAAGGAGGATAACCATATGTGTTGGGCAGCCGCAATACCTAT‑‑‑‑‑‑‑‑‑‑‑‑CGCTATATCTGGCGCTCAGGCTATCAGTGATCAGAACGCTCAGGCCAAAATGATTGCCGCTCAGACCGCTGCTGGTCGTCGTCAAGCTATGGAAATCATGAGGCCGACGAACATCCAGAATGCTGACCTATCGTTGCAAGCTCGAAGTGAACtctgcctctt                                                                                                                                                                          >  1:176755/4‑292 (MQ=255)`  
                                                                                                                                     `ggCAATACGTCCCACATTCGTTTCCTCAAGACTATCGGTGCGGTATTCCATGAAGAGTACACACGAGATGGTCAATTTCAGTTATTTACAAGCACGAAAGGAGGATAACCATATGTGTTGGGCAGCGGCAATACATAT‑‑‑‑‑‑‑‑‑‑‑‑CGCTATATCTGGCGCTCAGGCTATCAGTGATCAGAACGCTCAGGCCAAAATGATAGCCGCTCAGACCGCTGCTGGTCGTCGTCAAGCTATGGAAATCATGAGGCACACGAACATACAGAATGCTGAACTACCGTTGCCAGCTCGAAGTAAACTTGAGGAAGCg                                                                                                                                                                         >  1:269897/1‑301 (MQ=255)`  
                                                                                                                                           `aCGTCCCACATTCGTTTCCTCAAGACTATCGGTGCGGTATTCCATGAAGAGTACACACGAGATGGTCAATTTCAGTTATTTACAATCACGAAAGGAGGATAACCATATGTGTTGGGCAGCCGCAATACCTAT‑‑‑‑‑‑‑‑‑‑‑‑CGCTATATCTGGCGCTCAGGCTATCAGTGATCAGAACGCTCAGGCCAAAATGATTGCCGCTCAGACCGCTGCTGGTCGTCGTCAAGCTATGGAAATCATGAGGCAGACGAACATCCAGAATGCTGACCTATCGTTGCAAGCTCGAAGTAAACTTGAGGAAGCGTccgcc                                                                                                                                                                   >  1:206843/1‑301 (MQ=255)`  
                                                                                                                                                    `aTTCGTTTCCTCAAGACTATCGGTGCGGTATTCCATGAAGATTACACACGAGATGGTCAATTTCAGTTATTTACAATCACGAAAGGAGGATAACCATATGTGTTGGGCAGCCGCAATACCTAT‑‑‑‑‑‑‑‑‑‑‑‑CGCTATATCTGGCGCTCAGGCTATCAGTGATCCGAACGCTCAGGCCAAAATGATTGCCGCTCAGACCGCTGCTGGTCGTCGTCAAGCTATGGAAATCATGAGGCAGACGAACATCCAGAATGCTGACCTATCGTTGCAAGCTCGAAGTAAACTTAAGGAAGCGTCCGCCGAGTTGAcc                                                                                                                                                          <  1:7768/301‑1 (MQ=255)`  
                                                                                                                                                       `cGTTTCCTCAAGACTCTCGGTGCGGTATTCCATGAAGAGTACACACGAGATGTTCAATTTCAGTTATTTACAATAAAGAAAGGAGGATAACCATATGTGTTGGGCAGCCGCAATACCTAT‑‑‑‑‑‑‑‑‑‑‑‑CGCTATATCTGGCGCTCAGGCTATAAGTGATCAGAACGCTCAGGCCAAAATGATTGCCGCTCAGACCGCTGCTGGTAGTCGTCAAGCTATGGAAATCATGAGGCAGACGAACATCAAGAATGCTGACCTATCGTTGCAAGCTCGAAGTAAAGTTGAGGAAGCGTCCTCCGAGTTgcacccc                                                                                                                                                       >  1:245114/1‑295 (MQ=255)`  
                                                                                                                                                       `cGTTTCCTCAAGACTATCGGTGCGGTATTCCATGAAGAGTACACACGAGATGGTCAATTTCAGTTATTTACAATCCCGAAAGGAGGATAACCATATGTGTTGGGCAGCCGCAATACCTAT‑‑‑‑‑‑‑‑‑‑‑‑CGCTATATCTGGCGCTCAGGCTATCAGTTATCCGAACGCTAAGTCCAACATGATTGCCGCTCAGACCGCTGCTGGTAGTCGTCAAGCTCTGGAAATCATGAGGCAGACGAACATCCAGAAGGCTGACCTATCATTGCAAGCTCGCAGTAAACTTGAGGACACGTCCTCCGAGTTGACCTcc                                                                                                                                                       >  1:82396/1‑300 (MQ=255)`  
                                                                                                                                                            `ccTCAAGACTATCGGTGCGGTATTCCATGAAGAGTACACACGAGATGGTCAATTTCAGTTATTTACAATCACGAAAGGAGGATAACCATATGTGTTGGGCAGCCGCAATACCTAT‑‑‑‑‑‑‑‑‑‑‑‑CGCTATATCTGGCGCTCAGGCTATCAGTGATCAGAACGCTCAGGCCAAAATGATTGCCGCTCAGACCGCTGCTGGTCGTCGTCAAGCTATGGAAATCATGAGGCAGACGAACATCCAGAATGCTGACCTATCGTTGCAAGCTCGAAGTAAACTTGAGGGAGCGTCCGCCGAGTTGACCTcctgtct                                                                                                                                                  >  1:371655/1‑295 (MQ=255)`  
                                                                                                                                                            `ccTCAAGACTATCGGTGCGGTATTCCATGAAGAGTACACACGAGATGGTCAATTTCAGTTATTTACAATCACGAAAGGAGGATAACCATATGTGTTGGGCAGCCGCAATACCTAT‑‑‑‑‑‑‑‑‑‑‑‑CGCTATATCTGGCGCTCAGGCTATCAGTGATCAGAACGCTCAGGCCAAAATGATTGCCGCTCAGACCGCTGCTGGTCGTCGTCAAGCTATGGAAATCATGAGGCAGACGAACATCCAGAATGCTGACCTATCGTAGCAAGCTCGAAGTAAACTTGAGGAAGCGTCCGCCGAGTTGACCCCACAGaa                                                                                                                                                  >  1:320198/1‑301 (MQ=255)`  
                                                                                                                                                             `cTCAAGACTATCGGTGCGGTATTCCATGAAGAGTACACACGAGATGGTAAATTTCAGTTATTTACAATCACGAAAGGAGGATAACCATATGTGTTGGGCAGCCGCAATACCTAT‑‑‑‑‑‑‑‑‑‑‑‑CGCTATATCTGGCGCTCAGGCTATCAGTGATCAGAACGCTCAGGCAAAAATGATTGCCGCTCAGACCGCTGCTGGTCGTCGTCAAGCTATGGAAATCATGAGGCAGACGAACATCCAGAATGCTGACCTATCGTTGCAAGCTCGAAGTAAACTTGAGGAAGCGTCCGCCGAGTTGACCTCACAGAAc                                                                                                                                                 <  1:359615/301‑1 (MQ=255)`  
                                                                                                                                                                  `gACGATCGGTGCGGTATTCCATGAAGAGTACACACGAGATGGTCAATTTCAGTTATTTACAATCACGAAAGGAGGATAACCATATGTGTTGGGCAGCCGCAATACCTAT‑‑‑‑‑‑‑‑‑‑‑‑CGCTATATCTGGCGCTCAGGCTATCAGTGATCAGAACGCTCAGGCCAAAATGATTGCCGCTCAGACCGCTGCTGGTCGTCGTCAAGCTATGGAAAACATGAGGCAGACGAACATCCAGAATGCTGACCTATCGTTGCAAGCTCGAAGTAAACTTGAGGAAGCGTCCGCCGAGTTGACCCCACAGAACATGCa                                                                                                                                            <  1:171515/301‑1 (MQ=255)`  
                                                                                                                                                                   `ggctcggagatgtgtataagagacaggTACACACGAGATGGTCAATTTCAGTTATTTACAATCACGAAAGGAGGATAACCATATGTGTTGGGCAGCCGCAATACCTAT‑‑‑‑‑‑‑‑‑‑‑‑CGCTATATCTGGCGCTCAGGCTATCAGTGATCAGAACGCTCAGGCCAAAATGATTGCCGCTCAGACCGCTGCTGGTCGTCGTCAAGCTATGGAAATCATGAGGCAGACGAACATCCAGAATGCTGACCTATCGTTGCAAGCTCGAAGTAAACTTGAGGAAGCGTCCGCCGAGTTGACCACACAGAACATGCAg                                                                                                                                           <  1:29587/275‑1 (MQ=255)`  
                                                                                                                                                                    `cTATCGGTGCGGTATTCCATGAAGAGTACACACGAGATGGTCAATTTCAGTTATTTACAATCACGAAAGGAGGATAACCATATGTGTTGGGCAGCCGCAATACCTAT‑‑‑‑‑‑‑‑‑‑‑‑CGCTATATCTGGCGCTCAGGCTATCAGTGATCAGAACGCTCAGGCCAAAATGATTGCCGCTCAGACCGCTGCTGGTCGTCGTCAAGCTATGGAAATCATGAGGCAGACGAACATCCAGAATGCTGACCTATCGTTGCAAGCTCGAAGTAAACTTGAGGAAGCGTCCGCCGAGTTGACCTCCCAGCACATTCAGa                                                                                                                                          >  1:36514/1‑301 (MQ=255)`  
                                                                                                                                                                      `aTCGGTGCGGTATTCCATGAAGAGTACACACGAGATGGTCAATTTCAGTTATTTACAATCACGAAAGGAGGATAACCATATGTGTTGGGCAGCCGCAATACCTAT‑‑‑‑‑‑‑‑‑‑‑‑CGCTATATCTGGCGCTCAGGCTATCAGTGATCAGAACGCTCAGGCCAAAATGATTGCCGCTCAGACCGCTGCTGGTCGTCGTCAAGCTATGGAAATCATGAGGCAGACGAACATCCAGAATGCTGACCTATCGTTGCAATCTCGAAGTAAACTTGAGGAAGCGTCCGCCGAGTTGACCTCACAGAACATGCAgctg                                                                                                                                        >  1:91766/1‑298 (MQ=255)`  
                                                                                                                                                                      `aTCGGTGCGGTATTCCATGAAGAGTACACACGAGATGGTCAATTTCAGTTATTTACAATCACGAAAGGAGGATAACCATATGTGTTGGGCAGCCGCAATACCTAT‑‑‑‑‑‑‑‑‑‑‑‑CGCTATATCTGGCGCTCAGGCTATCAGTGATCAGAACGCTCAGGCCAAAATGATTGCCGCTCAGACCGCTGCTGGTCGTCGTCAAGCTATGGAAATCATGAGGCAGACGAACATCCAGAATGCTGACCTATCGTTGCAAGCTCGAAGTAAACTTGAGGAAGCGTCCGCCTAGTTGACCCCACAGAACATGCAGAAg                                                                                                                                        >  1:374964/1‑301 (MQ=255)`  
                                                                                                                                                                       `tCGGTGCGGTATTCCATGAAGAGTACACACGAGATGGTCAATTTCAGTTATTTACAATCACGAAAGGAGGATAACCATATGTGTTGGGCAGCCGCAATACCTAT‑‑‑‑‑‑‑‑‑‑‑‑CGCTATATCTGGCGCTCAGGCTATCAGTGATCAGAACGCTCAGGCCAAAATGATTGCCGCTCAGACCGCTGCTGGTCGTCGTCAAGCTATGGAAATCATGAGGCAGACGAACATCCAGAATGCTGACCTATCGTTGCAAGCTCGAAGTAAACTTGAGGAAGCGTCCGCCGAGTTGACCCCACAGAACATGCAgctgt                                                                                                                                       >  1:60886/1‑297 (MQ=255)`  
                                                                                                                                                                          `gTGCTGTATTCCATGAAGAGTACACACGAGATGGTCAATTTCAGTTATTTACAATCACGAAAGGAGGATAACCATATGTGTTGGGCAGCCGCAATACCTAT‑‑‑‑‑‑‑‑‑‑‑‑CGCTATATCTGGCGCTCAGGCTATCAGTGATCAGAACGCTCAGGCCAAAATGATTGCCGCTCAGACCGCTGCTGGTCGTCGTCAAGCTATGGAAATCATGAGGCAGACGAACATCCAGAATGCTGACCTATCGTTGCAAGCTCGAAGCAAACTTGAGGAAGCGTCCGCCGAGTTGACCCCACAGAACATGCAGAAGGTcc                                                                                                                                    >  1:329325/1‑301 (MQ=255)`  
                                                                                                                                                                            `gCGGTATTCCATGAAGAGTACACACGAGATGGTCAATTTCAGTTATTTACAATCACGAAAGGAGGATAACCATATGTGTTGGGCAGCCGCAATACCTATCGCTATATCTGGCGCTATATCTGGCGCTCAGGCTATCAGTGATCAGAACGCTCAGGCCAAAATGATTGCCGCTTAGACCGCTGCTGGTTGTCGTCAAGCTATGGAAATCATGAGACAGACGAACATCCAGAATGCTGACCAATCGTTGCAAGCTCGAAGTAAACTAGAGGAAGCGTCCGCCGAGTTGACCGCACAGAACacg                                                                                                                                              >  1:384525/1‑299 (MQ=255)`  
                                                                                                                                                                            `gCGGTATTCCATGAAGAGTACACACGAGATGGTCAATTTCAGTTATTTACAATCACGAAAGGAGGATAACCATATGTGTTGGGCAGCCGCAATACCTAT‑‑‑‑‑‑‑‑‑‑‑‑CGCTATATCTGGCGCTCAGGCTATCAGTGATCAGAACGCTCAGGCCAAAATGATTGCCGCTCAGACCGCTGCTGGTCGTCGTCAAGCTATGGAAATCATGAGGCAGACGAACATCCAGAATGCTGACCTATCGTTGCAAGCTCGAAGTAAACTTGAGGAAGCGTCCGCCGAGTTGACCTCACAGAACATGCAGAAGGTCCaa                                                                                                                                  >  1:134339/1‑301 (MQ=255)`  
                                                                                                                                                                            `gCGGTATTCCATGAAGAGTACACACGAGATGGTCAATTTCAGTTATTTACAATCACGAAAGGAGGATAACCATATGTGTTGGGCAGCCGCAATACCTAT‑‑‑‑‑‑‑‑‑‑‑‑CGCTATATCTGGCGCTCAGGCTATCAGTGATCAGAACGCTCAGGCCAAAATGATTGCCGCTCAGACCGCTGCTGGTCGTCGTCAAGCTATGGAAATCATGAGGCAGACGAACATCCAGAATGCTGACCTATCGTTGCAAGCTCGAAGTAAACTTGAGGAAGCGTCCGCCGAGTTGACCCCACAGAACATGCAGAAGGTCCaa                                                                                                                                  >  1:193554/1‑301 (MQ=255)`  
                                                                                                                                                                            `gCGGTATTCCATGAAGAGTACACACGAGATGGTCAATTTCAGTTATTTACAATCACGAAAGGAGGATAACCATATGTGTTGGGCAGCCGCAATACCAAT‑‑‑‑‑‑‑‑‑‑‑‑CGCTATATCTGGCGCTCAGGCTATCAGTGATCAGAACGCTCAGGCCAAAATGATTGCCGCTCAGCCCGCTGCTGGTCGTCGTCAAGCTATGGAAATCATGAGGCAGACGAACATCAAGACTGCTGACCTATCGTTGCAAGCTCGAAGTAAACTTGAGGAAACGTCCTCCGAGTTGACCTCACAGAACATCCAGAAGGTCCaa                                                                                                                                  >  1:497610/1‑301 (MQ=255)`  
                                                                                                                                                                              `ggTATTCCATGAAGAGTACACACGAGATGGTCAATTTCAGTTATTTACAATCACGAAAGGAGGATAACCATATGTGTTGGGCAGCCGCAATACCTAT‑‑‑‑‑‑‑‑‑‑‑‑CGCTATATCTGGCGCTCAGGCTATCAGTGATCAGAACGCTCAGGCCAAAATGATTGCCGCTCAGACCGCTGCTGGTCGTCGTCAAGCTATGGAAATCATGAGGCAGACGAACATCCAGAATGCTGACCTATCGTTGCAAGCTCGAAGTAAACTTGAGGAAGCGTCCGCCGAGTTGACCCCACAGAACATTCAGAAGGTCCAAGc                                                                                                                                >  1:341528/1‑301 (MQ=255)`  
                                                                                                                                                                              `ggTATTCCATGAAGAGTACACACGAGATGGTCAATTTCAGTTATTTACAATCACGAAAGGAGGATAACCATATGTGTTGGGCAGCCGCAATACCTAT‑‑‑‑‑‑‑‑‑‑‑‑CGCTATATCTGGCGCTCAGGCTATCAGTGATCAGAACGCTCAGGCCAAAATGATTGCCGCTCAGACCGCTGCTGGTCGTCGTCAAGCTAAGGAAATCATGAGGCAGACGAACATCCAGAATGCTGACCTATCGTTGCAAGCTCGAAGTAAACTTGAGGAAGCGTCCGCCGAGTTGACCTCACAGAACATGCAGAAGGTCCAAGc                                                                                                                                <  1:426570/301‑1 (MQ=255)`  
                                                                                                                                                                                `tATTCCATGAAGAGTACACACGAGATGGTCAATTTCAGTTATTTACAATCACGAAAGGAGGATAACCATATGTGTTGGGCAGCCGCAATACCTAT‑‑‑‑‑‑‑‑‑‑‑‑CGCTATATCTGGCGCTCAGGCTATCAGTGATCAGAACGCTCAGGCCAAAATGATTGCCGCTCAGACCGTTGCTGGTCGTCGTCAAGCTATGGAAATCATGAGGCAGACGAACATCCAGAATGCTGACCTATCGTTGCAAGCTCGAAGTAAACTTGAGGAAGCGTCCGCCGAGTTGACCTCACAGAACATGCAGAAGGTCCAAGCTa                                                                                                                              <  1:233262/301‑1 (MQ=255)`  
                                                                                                                                                                                 `aTTCCATGAAGAGTACACACGAGATGGTCAATTTCAGTTATTTACAATCACGAAAGGAGGATAACCATATGTGTTGGGCAGCCGCAATACCTAT‑‑‑‑‑‑‑‑‑‑‑‑CGCTATATCTGGCGCTCAGGCTATCAGTGATCAGAACGCTCAGGCCAAAATGATTGCCGCTCAGACCGCTGCTGGTCGTCGTCCAGCTATGGAAATCATGCGGCAGACGAACATCCAGAATGCTGACCTATCGTTGCACGCTCGAAGTAAACTGGAGGAAGCGTCCGCAGAGTTGACCCCCCAGAACATGCAgctgtctcttatcca                                                                                                                             >  1:159637/1‑287 (MQ=255)`  
                                                                                                                                                                                 `aTTCCATGAAGAGTACACACGAGATGGTCAATTTCAGTTATTTACAATCACGAAAGGAGGATAACCATATGTGTTGGGCAGCCGCAATACCTAT‑‑‑‑‑‑‑‑‑‑‑‑CGCTATATCTGGCGCTCAGGCTATCAGTGATCAGAACGCTCAGGCCAAAATGATTGCCGCTCAGACCGCTGCTGGTCGTCGTCAAGCTATGGAAATCATGAGGCAGACGAACATCCAGAATGCTGACCTATCGTTGCAAGCTCGAAGTAAACTTGAGTAAGCGTCCGCCGATTTGACCTCCCAGAACATGCAGAAGGTCCAAGcact                                                                                                                             >  1:398483/1‑298 (MQ=255)`  
                                                                                                                                                                                   `tCCATGAAGAGTACACACGAGATGGTCAATTTCAGTTATTTACAATCACGAAAGGAGGATAACCATATGTGTTGGGCAGCCGCAATACCTAT‑‑‑‑‑‑‑‑‑‑‑‑CGCTATATCTGGCGCTCAGGCTATCAGTGATCAGAACGCTCAGGCCAAAATGATTGCCGCTCAGACCGCTGCTGGTCGTCGTCAAGCTATGGAAATCATGAGGCAGACGAACATCCAGAATGCTGACCTATCGTTGCAAGCTCGAAGTAAACTTGAGGAAGCGTCAGCCGAGTTGACCCCACAGAACATGCAGAAGGTCCAAGCTATTg                                                                                                                           >  1:337620/1‑301 (MQ=255)`  
                                                                                                                                                                                    `ccATGAAGAGTACACACGAGATGGTCAATTTCAGTTATTTACAATCACGAAAGGAGGATAACCATATGTGTTGGGCAGCCGCAATACCTAT‑‑‑‑‑‑‑‑‑‑‑‑CGCTATATCTGGCGCTCAGGCTATCAGTGATCAGAACGCTCAGGCCAAAATGATTGCCGCTCAGACCGCTGCTGGTAGTCGTCAAGCTATGGAAATCATGAGGCAGACGAACATCCAGAATGCTGACCTATCGTTGCAAGCTCGAAGTACACTTGAGGAAGCGTCCGCCGAGTTGACCTCACAGAAAATGCAGAAGGTCCAAGCTATTgg                                                                                                                          >  1:260256/1‑301 (MQ=255)`  
                                                                                                                                                                                       `tgaaaggtaccaacGGGATGAGCAATTTCAGTTAATTACAATCACGAAAGGAGGATAACCATATGTGTTGGGCAGCCGCAATATCTAT‑‑‑‑‑‑‑‑‑‑‑‑CGCTATATCTGGCGCTCAGGCTATCAGTGATCAGAACGCTCAGGCCAAAATGTATGCCGCTCAGACCGATGCTGGTCGTCGTCAAGCTATGGAAAACATGAGGCAGACGAACATCCAGAATGCTGACCTATCGTTGCAAGCTCGAAGTAAACTTGAGGAAGCGTCCGCCGAGTTGACCCCACAGAACATGCAGAAGGTCCAAGCTATTGGGTc                                                                                                                       <  1:508393/289‑1 (MQ=255)`  
                                                                                                                                                                                       `tGAAGAGTACAAACGAGATGGTCAATTTCAGTTATTTACAATCACGAAAGGAGGATAACCATATGTGTTGGGCAGCCGCAATACCTAT‑‑‑‑‑‑‑‑‑‑‑‑CGCTATATCTGGCGCTCAGGCTATCAGTGATCAGAACGCTCAGGCCAAAATGATTGCCGCTCAGACCGCTGCTGGTCGTCGTCAAGCTATGGAAATCATGAGGCAGACGAACATCCAGAATGCTGACCTATCGTTGCAAGCTCGAAGTAAACTTGAGGAAGCGTCCGCCGAGTTGACCTCACAGAACATGCAGAAGGTCCAAGCTATTGGGTc                                                                                                                       <  1:24156/301‑1 (MQ=255)`  
                                                                                                                                                                                           `gagTACACACGAGATGGTCAATTTCAGTTATTTACAATCACGAAAGGAGGATAACCATATGTGTTGGGCAGCCGCAATACCTAT‑‑‑‑‑‑‑‑‑‑‑‑CGCTATATCTGGCGCTCAGGCTATCAGTGATCAGAACGCTCAGGCCAAAATGATTGCCGCTCAGACCGCTGCTGGTCGTCGTCAAGCTATGGAAATCATGAGGCAGACGAACATCCAGAATGCTGACCTATCGTTGCAAGCTCGAAGTAACCTTGAGGAAGCGTCCGCCGAGTTGACCCCACAGAACATGCAGAAGGTCCAAGCTATTGGGTCTATc                                                                                                                   >  1:435673/1‑301 (MQ=255)`  
                                                                                                                                                                                             `gTACACACGAGATGGTCAATTTCAGTTATTTACAATCACGAAAGGAGGATAACCATATGTGTTGGGCAGCCGCAATACCTAT‑‑‑‑‑‑‑‑‑‑‑‑CGCTATATCTGGCGCTCAGGCTATCAGTGATCAGAACGCTCATGCCAAAATGATTGCCGCTCAGACCGCTGCTGGTCGTCGTCAAGCTATGGAAATCATGAGGCAGACGAACATCCAGAATGCTGACCTATCGTTGCAAGCTCGAAGTAAACTTGAGGAAGCGTCCGCCGAGTTGACCTCACAGAACATGCAGAAGGTCCAAGCTATTGGGTCTATCCg                                                                                                                 >  1:255015/1‑301 (MQ=255)`  
                                                                                                                                                                                             `gTACACACGAGATGGTCAATTTCAGTTATTTACAATCACGAAAGGAGGATAACCATATGTGTTGGGCAGCCGCAATACCTAT‑‑‑‑‑‑‑‑‑‑‑‑CGCTATATCTGGCGCTCAGGCTATCAGTGATCAGAACGCTCAGGCCAAAATGATTGCCGCTCAGACCGCTGCTGGTCGTCGTCAAGCTATGGAAATCATGATGCAGACGAACATCCAGAATGCTGACCTATCGTTACAAGCTCGAAGTAAACTTGAGGAAGCGTCCGCCGAGTTGACCCCACAGAACATGCAGAAGGTCCAAGCTATTGGGTCTATCCg                                                                                                                 >  1:355478/1‑301 (MQ=255)`  
                                                                                                                                                                                             `gTACACACGAGATGGTCAATTTCAGTTATTTACAATCACGAAAGGAGGATAACCATATGTGTTGGGCAGCCGCAATACCTAT‑‑‑‑‑‑‑‑‑‑‑‑CGCTATATCTGGCGATCAGGCTATCAGTGATCAGAACGCTCAGGCCAAAATGATTGCCGCTCAGACCGATGCTGGTCGTCGTCAAGCTATGGAAATCATGAGGCAGACGAAAATCCAGAATGCTGACCTATCGTTGCAAGAACGAAGTAAACTTGAGGAAGCGTACGACGGGATGAACACAAAGAAAATGCAGAAGGGACAAGCAATTgctggcgctta                                                                                                                 >  1:315372/1‑291 (MQ=255)`  
                                                                                                                                                                                             `ctcggagatgtgtataagagtcaggTTATTTACACTCACGAAAGGAGGATAACCATATGTGTTGGGCAGTCGCAATACCTAT‑‑‑‑‑‑‑‑‑‑‑‑CGCTATATCTGGCGCTCAGGCTATCAGTGATCAGAACGCTCAGGCCAAAATGATTGCCGATCAGACCGCTGCTGGTCGTCGTCAAGCTATGGAAATCATGAGGCAGACGAACATCCAGAATGCTGACCTATCGTTGCAAGCTCGAAGTAAACTTGAGGAAGCGTCCGCCGAGTTGACCCCACAGAACATGCAGAAGGTCCAAGCTATTGGGTATATCCg                                                                                                                 <  1:64535/277‑1 (MQ=255)`  
                                                                                                                                                                                                             `cAATTTCAGTTATTTACAATCACGAAAGGAGGATAACCATATGTGTTGGGCAGCCGCAATACCTAT‑‑‑‑‑‑‑‑‑‑‑‑CGCTATATCTGGCGCCCAGGCTATCAGTGATCAGAACGCACAGGCCAAAATGATTGCCGCTCAGACCGCTGCTGGTCGTCGTCAAGCTATGGAAATCATGAGGCAGACGAACATCCAGAATGCTGACCTATCGTTGCAAGCTCGAAGGAAACTTGAGGAAGAGTCAGCCGAGTTGACCCCACAAAAAATGCAGAAGGTCCAAGCTATTGGGTCTAACAGAGCGGCTATAGgagag                                                                                                 >  1:311684/1‑301 (MQ=255)`  
                                                                                                                                                                                                             `cAATTTCAGTTATTAAAAAGCACGAAAGGAGGATAACCATATGTGTTGGGCAGCCGCAATACCTAT‑‑‑‑‑‑‑‑‑‑‑‑CGCTATATCGGGCGCTCAGGCTATCAGTGATCAGAACGCTCAGGCCAAAATGATTGCCGCTCAGACCGCTGCTGGTCGTCGTCAAGCTATGGAAATCATGAGGCAGACGAACATCCAGAATGCTGACCTATCGTTGCAAGCTCGAAGTAAACTTGAGGAAGCGTCCGCCGAGTTGACCCCACAGAACATGCAGAAGGTCCAAGCTATTGGGTCTATCCGAGCGGCTATCGgagag                                                                                                 <  1:96138/301‑1 (MQ=255)`  
                                                                                                                                                                                                             `aaaTGTCAGTTATTTACAATCACGAAAGGAGGATAACCATATGTGTTGGGCAGCCGCAATACCTAT‑‑‑‑‑‑‑‑‑‑‑‑CGCTATATCTGGCGCTCAGGCTATCAGTGATCAGAACGCTCAGGCCAAAATGATTGCCGCTCAGACCGCTGCTGGTCGTCGTCAAGCTATGGAAATCATGAGGCAGACGAACATCCAGAATGCTGACCTATCGTTGCAAGCTCGAAGTAAACTTGAGGAAGCGTCCGCCGAGTTGACCTCACAGAACATGCAGAAGGTCCAAGCTATTGGGTCTATCCGAGCGGCTATCGgagag                                                                                                 <  1:219414/300‑1 (MQ=255)`  
                                                                                                                                                                                                                `tttCAGTTATTTACAATCACGAAAGGAGGATAACCATATGTGTTGGGCAGCCGCAATACCTAT‑‑‑‑‑‑‑‑‑‑‑‑CGCTATATCTGGCGCTCAGGCTATCAGTGATCAGAACGCTCAGGCAAAAATGATTGCCGCTCAGACCGCTGCTGGTCGTCGTCAAGCTATGGAAATCATGAGGCAGACGAACATCCAGAATGCTGACCTAGCGTTGCAAGCTCGAAGTAAACTTGAGGAAGCGTCTGCCGAGTTGACCTCACAGAACATGCAGAAGGTACAAGCTATTGGGTCTATCCGAGCGGCTATCGGAGAGAGt                                                                                              >  1:290603/1‑301 (MQ=255)`  
                                                                                                                                                                                                                     `gTTATTTACAATCACGAAAGGAGGATAACCATATGTGTTGGGCAGCCGCAATACCTAT‑‑‑‑‑‑‑‑‑‑‑‑CGCTATATCTGGCGCTCAGGCTATCAGTGATCAGAACGCTCAGGCCAAAATGATTGCCGCTCAGACAGCTGCTGGTCGTCGTCAAGCTATGGAAATCATGAGGCAGACGAACATCCAGAATGCTGACCTATCGTTGCAAGCTCGAAGTAAACTTGAGGAAGCGTCCGCCGAGTTGACCCCACAGAACATGCAGAAGGTCCAAGCTATTGGGTCTATCCGAGCGGCTATCGGAGAGAGTATGCt                                                                                         <  1:167448/301‑1 (MQ=255)`  
                                                                                                                                                                                                                        `aTTTACAATCACGAAAGGAGGATAACCATATGTGTTGGGCAGCCGCAATACCTAT‑‑‑‑‑‑‑‑‑‑‑‑CGCTATGTCTGGCGCTCAGGCTATCAGTGATCAGAACGCTCAGGCCAAAATGATTGCCGCTCAGACCGCTGCTGGTCGTCGTCAAGCTATGTAAATCATGAGGCAGACGAACATCCAGAATGCTGACCTATCGTTGCAAGCTCGAAGTAAACTTGAGGAAGCGTCCGCCGAGTTGACCCCACAGAACATTCAGAAGGTCCAAGCTATTGGGTCTATCCGAGCGGCTATCGGAGAGAGTactgtctc                                                                                      >  1:180500/1‑294 (MQ=255)`  
                                                                                                                                                                                                                           `tACAATCACGAAAGGAGGATAACCATATGTGTTGGGCAGCCGCAATACCTAT‑‑‑‑‑‑‑‑‑‑‑‑CGCTATATCTGGCGCTCAGGCTATCAGTGATCAGAACGCTCAGGCCAAAATGATTGCCGCTCAGACCGCTGCTGGTCGTCGTCAAGCTATGGAAATCATGAGGCAGACGAACATCCAGAATGCTGACCTATCGTTGCAAGCTCGAAGTAAACTTGAGGAAGCGTCCGCCGAGTTGACCTCACAGAACATGCAGAAGGTCCAAGCTATTGGGTCTATCCGAGCGGCTATCGGAGAGAGTATGCTTGAAgg                                                                                   <  1:477020/301‑1 (MQ=255)`  
                                                                                                                                                                                                                            `aCAATCACGAAAGGAGGAGAACCATATGTGTTGGGCAGCCGCAATACCTAT‑‑‑‑‑‑‑‑‑‑‑‑CGCTATATCTGGCGCTCAGGCTATCAGTGATCAGAACGCTCAGGCCAAAATGATTGCCGCTCAGGCCGCTGCTGGTCGTCGTCAAGCTATGGAAATCATGAGGCAGACGAACATCCAGAATGCTGACCTATCGTTGCAAGCTCGAAGTAAACTTGAGGAAGCGTCCGCCGAGTTGACCCCACAGAACATGCAGAAGGTCCAAGCTATTGGGTCTATCCGAGCGGCTATCGGAGAGAGTATGCTTGAAGGt                                                                                  <  1:123902/301‑1 (MQ=255)`  
                                                                                                                                                                                                                                 `cACGAAAGGAGGATAACCATATGTGTTGGGCAGCCGCAATACCTCT‑‑‑‑‑‑‑‑‑‑‑‑CGCTATATCTGGCGCTCAGGCTATCAGTGATCAGAACGCTCAGGCCAAAATGATTGCCGCTCAGACCGCTGCTGGTCGTCGTCAAGCTATGGAAATCATGAGGCAGACGAACATCCAGAATGCTGACCTATCGTTGCAAGCTCGAAGTAAACATGAGGAAGCGTCCGACGAGTTGACCTCACGGTACATGCAGAAGGTCCAAGCTAATGGGGCTATCCGAGCGGCTATCGGAGAGAGTATGCTTGAAGGTTCCta                                                                             >  1:210893/1‑300 (MQ=255)`  
                                                                                                                                                                                                                                 `cACGAAAGGAGGATAACCATATGTGTTGGGCAGCCGCAATACCTAT‑‑‑‑‑‑‑‑‑‑‑‑CGCTATATCTGGCGCTCAGGCTATCAGTGATCAGAACGCTCAGGCTAAAATGATTGCCGCTCAGACCGCTGCTGGTCGTCGTCAAGCTATGGAAATCATGAGGCAGACGAACATCCAGAATGCTGACCTATCGTTGCAAGCTCGAAGTAAACTTGAGGAATCGTCCGCCGAGTCGACCCCACAGCACATGCAGAAGGCCCAAGCTATTGGGTCAATCCGAGCGGCTATCGGAGAGAGTATGCTTGAAGGttcttc                                                                             >  1:138900/1‑301 (MQ=255)`  
                                                                                                                                                                                                                                 `cACGAAAGGAGGATAACCATATGTGTTGGGCAGCCGCAATACCTAT‑‑‑‑‑‑‑‑‑‑‑‑CGCTATATCTGGAGCTCAGTCTATCAGTGATCAGAACGCTCAGTCCAAAATGATTGCCTCTCAGACCGCTGCTGGTCGTCGTCATGCTATGGAAATCATGTGGCAGACTAACATCCAGAATGCTGACCTATCGTTGCAATCTAGAAGTAAACTTGAGGAAGCGTCCGCCGAGTTGACCCCACAGAACATGCAGAAGGTCCAAGCTATTGGGTCTATCCGCGAGGCTATCGGAGAGAGTATGCTTGAAGTTTCCTc                                                                             >  1:286226/1‑301 (MQ=255)`  
                                                                                                                                                                                                                                      `gacagggaTAACCATATGTGTTGGGCAGCCGCAATACCTAT‑‑‑‑‑‑‑‑‑‑‑‑CGCTATATCTGGCGCTCAGGCTATCAGTGATCAGAACGCTCAGGCCAAAATGATTGCCGCTCAGACCGCTGCTGGTCGTCGTCAAGCTATGGAAATCATGAGGCAGACGAACATCCAGAATGCTGACCTATCGTTGCAAGCTCGAAGTAAACTTGAGGAAGCGTCCGCCGAGTTGACCCCACAGAACATGCAGAAGGTCCAAGCTATTGGGTCTATCCGAGCGGCTATCGGAGAGAGTATGCTTGAAGGTTCCTCAATgg                                                                        <  1:69227/296‑1 (MQ=255)`  
                                                                                                                                                                                                                                        `tgaggaTAACCATATGTGTTGTGCAGCCGCAATACCTAT‑‑‑‑‑‑‑‑‑‑‑‑CGCTATATCTGGCGCACAGGCTATCAGTGATCAGAACGCTCAGGCCAAAATGATTGCCGCTCAGACCGCTGCTGGTCGTCGTCAAGCTATGGAAATCATGAGGCAGACGAACATCCAGAATGCTGACCTATCGTTGCAAGCTCGAAGTAAACTTGAGGAAGCGTCCGCCGAGTTTACCCCACAGAACATGCAGAAGGTCCAAGCTATTGGGTCTATCCGAGCGGCTATCGGAGAGAGTATGCTTGAAGGTTCCTCAATGGAc                                                                      <  1:53028/300‑1 (MQ=255)`  
                                                                                                                                                                                                                                        `ggaggaTAACCATATGTGTGGGGCAGGCGCAATACCTAT‑‑‑‑‑‑‑‑‑‑‑‑CGCTATATCTGGCGCTCAGGCTATCAGTGAACAGAACGATCAGGCCAAAATGATTGCCGCTCAGACCGCTGCTGGTAGTCGTCAAGCTATGGAAATCATGAGGCAGACGAACATCCAGAATGCTGACCTATCGTTGCAAGCTCGAAGTAAACTTGAGGAAGCGTCCGCCGAGTTGACCTCACAGAACATGCAGAAGGTCCAAGCTATTGGGTCTATCCGAGCGGCTATCTGAGAGAGTATGCTTGAAGGTTCCTCAATGGAc                                                                      <  1:114481/301‑1 (MQ=255)`  
                                                                                                                                                                                                                                         `gaggaTAACCATATGTGTTGGGCAGCCGCAATACCTAT‑‑‑‑‑‑‑‑‑‑‑‑CGCTATATCTGGCGCTCAGGCTATCAGTGATCAGAACGCTCAGGCCAAAATGATTGCCGCTCAGACCGCTGCTGGTCGTCGTCAAGCTATGGAAATCATGAGGCAGACGAACATCCAGAATGCTGACCTATCGTTGCAAGCTCGAAGTAAACTTGAGGAAGCGTCCGCAGAGTTGACCCCACAGAACATGCAGAAGGTCCAAGCTATTGGGTCTATCCGAGCGGCTATCGGAGAGAGTATGCTTGAAGGTTCCTCAATGGAcc                                                                     >  1:153535/1‑301 (MQ=255)`  
                                                                                                                                                                                                                                         `gaggaTAACCATATGTGTTGGGCAGCCGCAATACCTAT‑‑‑‑‑‑‑‑‑‑‑‑CGCTATATCTGGCGCTCAGGCTATCAGTGATCAGAACGCTCAGGACAAAATGATTGCCGCTCAGACCGCTGCTGGTCGTCGTCAAGCTATGGAAATCATGAGGCAGACGAACATCCAGAATGCTGACCTATCGTTGCAAGCTCGAAGTAAACTTGAGGAAGCGTCCGCCGAGTTGACCTCACAGAACATGCAGAAGGTCCAAGCTATTGGGTCTATCCGAGCGGCTATCGGAGAGAGTATGCTTGAAGGTTCCTCAATGGAcc                                                                     <  1:8702/301‑1 (MQ=255)`  
                                                                                                                                                                                                                                             `aTAACCATATGTGTTGGGCAGCCGCAATACCTAT‑‑‑‑‑‑‑‑‑‑‑‑CGCTATATCTGGCGCTCAGGCTATCAGTGATCAGAACGCTCAGGCCAAAATGATTGCCGCTAAGACCGCTGCTGGTCGTCGTCAAGCTATGGAAATCATGAGGCAGACGAACATCCAGAATGCTGACCTATCGTTGCAAGCTCGAAGTAAACTTGAGGAAGCGTCCGCCGAGTTGACCCCACAGAACATGCAGAAGGTCCAAGCTATTGGGTCTATCCGAGCGGCTATCGGAGAGAGTATGCTTGAAGGTTCCTCAATGGACCGCAt                                                                 <  1:265121/301‑1 (MQ=255)`  
                                                                                                                                                                                                                                              `tAAGCATATGTGTTGGGCGGCCGCAATACCTAT‑‑‑‑‑‑‑‑‑‑‑‑CGCTATATCTGGCGCTCAGGCTATCAGTGATCAGAACGCTCAGGCCAAAATGATTGCCGCTCAGACCGCTGCTGGTCGTCGTCAAGCTATGGAAATCATGAGGCAGACGAACATCCAGAATGCTGACCTATCGTTGCAAGCTCGAAGTAAACTTGAGGAAGCGTCCGCCGAGTTGACCCCACAGAACATGCAGAAGGTCCAAGCTATTGGGTCTATCCGAGCGGCTATCGGAGAGAGTATGCTTGAAGGTTCCTCAATGGACCGCAtt                                                                <  1:73370/301‑1 (MQ=255)`  
                                                                                                                                                                                                                                               `aaCCATATGTGTTGGGCAGCCGCAATACCTAT‑‑‑‑‑‑‑‑‑‑‑‑CGCTATATCTGGCGCTCAGGCTATCAGTGATCAGAACGCTCAGGCCAAAATGATTGCCGCTCAGACCGCTGCTGGTCGTCGTCAAGCTATGGAAATCATGAGGCAGACGAACATCCAGAATGCTGACCTATCGTTGCAAGCTCGAAGTAAACTTGAGGAAGCGTCCACCGAGTTGACCCCACAGAACATGCAGAAGGTCCAAGCTATTGGGTCTATCCGAACGGCTATCGGAGAGAGTATGCTTGAAGGTTCCTCAATGGACCGCATTa                                                               >  1:283248/1‑301 (MQ=255)`  
                                                                                                                                                                                                                                                `aCCATATGTGTTGGGCAGCCGCAATACCTAT‑‑‑‑‑‑‑‑‑‑‑‑CGCTATATCTGGCGCTCAGGCTATCAGTGATCAGAACGCTCAGGCCAAAATGATTGCCGCTCAGACCGCTGCTGGTCGTCGTCAAGCTATGGAAATCATGAGGCAGACGAACATCCAGAATGCTGACCTATCGTTGCAAGCTCGAAGTAAACTTGAGGAAGCGTCCGCCGAGTTGACCCCACAGAACATGCAGAAGGTCCAAGCTATTGGGTCTATCCGAGCGGCTATCGGAGAGAGTATGCTTGAAGGTTCCCCAATGGACCGCATTaa                                                              >  1:279261/1‑301 (MQ=255)`  
                                                                                                                                                                                                                                                 `ccATATGTGTTGGGCAGCCGCAATACCTAT‑‑‑‑‑‑‑‑‑‑‑‑CGCTATATCTGGCGCTCAGGCTATCAGTGATCAGAACGCTCAGGCCAAAATGATTGCCGCTCAGACCGCTGCTGGTCGTCGTCAAGCTATGGAAATCATGAGGCAGACGAACATCCAGAATGCTGACCTATCGTTGCAAGCTCGAAGTAAACTTGAGGAAGCGTCCGCCGAGTTGACCTCACAGAACATGCAGAAGGTCCAAGCTATTGGGTCTATCCGAGCGGCTATCGGAGAGAGTATGCTTGAAGGTTCCTCAATggctgtctcttat                                                             >  1:419940/1‑290 (MQ=255)`  
                                                                                                                                                                                                                                                   `atgtgtataagagacaggCAATCCCTAT‑‑‑‑‑‑‑‑‑‑‑‑CGCTATATCTGGCGCTCAGGCTATCAGTGATCAGAACGCTCAGGCCAAAATGATTGCCGCTCAGACCGCTGCTGGTCGTCGTCAAGCTATGGAAATAATGAGGCAGACGAACATCCAGAATGCTGACCTATCGTTGCAAGCTCGAAGTAAACTTGAGGAAGCGTCCGCCGAGTTGACCCCACAGAACATGCAGAAGGTCCAAGCTATTGGGTCTATCCGAGCGGCTATCGGAGAGAGTATGCTTGAAGGTTCCTCAATGGACCGCATTAAGCg                                                           <  1:197842/284‑1 (MQ=255)`  
                                                                                                                                                                                                                                                       `tataagagacagcGCAATACCTAT‑‑‑‑‑‑‑‑‑‑‑‑CGCTATATCTGGCGCTCAGGCTATCAGTGATCAGAACGCTCAGGCCAAAATGATTGCCGCTCAGACCGCTGCTGGTCGTCGTCAAGCTATGGAAATCATGAGGCAGACGAACATCCAGAATGCTGACCTATCGTTGCAAGCTCGAAGTAAACTTGAGGAAGCGTCCGCCGAGTTGACCCCACAGAACATGCAGAAGGTCCAAGCTATTTGGTCTATCCGAGCGGCTATCGGAGAGAGTATGCTTGAAGGTTCCTCAATGGACCGCATTAAGCGAGTc                                                       <  1:409986/289‑1 (MQ=255)`  
                                                                                                                                                                                                                                                            `gggCAGCCGCAATACCTAT‑‑‑‑‑‑‑‑‑‑‑‑CGCTATATCTTGCGCTCAGGCTATCAGTGATCAGAACGCTCAGGCCAAAATGATTGCCGCTCAGACCGCTGCTGGTCGTCGTCAAGCTATGGAAATCATGAGGCAGACGAACATCCAGAATGCTGTCCTATCGTTGCAAGCTCGAAGTAAACTTGAGGAAGCGTCCGCCGAGTTGACCTCACAGAACATGCAGAAGGTCCAAGCTATTGGGTCTATCCGAGCGGCTATCGGAGAGAGTATGCTTGAAGGTTCCTCAATGGACCGCATTAAGCGAGTCACAGa                                                  >  1:188243/1‑301 (MQ=255)`  
                                                                                                                                                                                                                                                                 `gCCGCAATACCTAT‑‑‑‑‑‑‑‑‑‑‑‑CGCTATATCTGGCGCTCAGGCTATCAGTGATCAGAACGCTCAGGCCAAAATGATTGCCGCTCAGACCGCTGCTGGTCGTCGTCAAGCTATGGAAATCATGAGGCAGACGAACATCCAGAATGCTGACCTATCGTTGCAAGCTCGAAGTAAACTTGAGGAAGCGTCCGCCGAGTTGACCTCACAGAACATGCAGAAGGTCCAAGCTATTGGGTCTATCCGAGCGGCTATCGGAGAGAGTATGCTTGAAGGTTCCTCAATGGACCGCATTAAGCgctgtctcttataca                                             >  1:462949/1‑287 (MQ=255)`  
                                                                                                                                                                                                                                                                 `gCCGCAATACCTAT‑‑‑‑‑‑‑‑‑‑‑‑CGCTATATCTGGCGCTCAGGCTATCAGTGATCAGAACGCTCAGGCCAAAATGATTGCCGCTCAGACCGCTGCTGGTCGTCGTCAAGCTATGGAAATCATGAGGCAGACGAACATCCAGAATGCTGACCTATCGTTGCAAGCTCGAAGTAAACTTGAGGAAGCGTCCGCCGAGTTGACCCCACAGAACATGCAGAAGGTCCAAGCTATTGGGTCTATCCGAGCGGCTATCGGAGAGAGTATGCTTGAAGGTTCCTCAATGGACCGCATTAAGCGAGTCCCAGAAGGAc                                             >  1:492021/1‑301 (MQ=255)`  
                                                                                                                                                                                                                                                                  `ccGCAATACCTAT‑‑‑‑‑‑‑‑‑‑‑‑CGCTATATCTGGCGCTCAGGCTATCAGTGATCAGAACGCTCAGGCCAAAATGATTGCCGCTCAGACCGCTGCTGGTCGTCGTCAAGCTATGGAAATCATGAGGCAGACGAACATCCAGAATGCTGACCTATCGTTGCAAGCTCGAAGTAAACTTGAGGAAGCGTCCGCCGAGTTGACCCCACAGAACATGCAGAAGGTCCAAGCTATTGGGTCTATCCGAGCGGCTATCGGAGAGAGTATGCTTGAAGGTTCCTCAATGGACCGCATTAAGCGAGTCACAGAAGGACa                                            >  1:128481/1‑301 (MQ=255)`  
                                                                                                                                                                                                                                                                       `aTACCTAT‑‑‑‑‑‑‑‑‑‑‑‑CGCTATATCTGGCGCTCAGGCTATCAGTGATCAGAACGCTCAGTCCAAAATGATTGCCGCTCAGACCGCTGCTGGTCGTCGTCAAGCTATGGAAATCATGAGGCAGACGAACATCCAGAATGCTGACCTATCGTTGCAAGCTCGAAGTAAACTTGAGGAAGCGTCCGCCGAGTTTACCCCACAGAACATGCAGAAGGTCCAAGCTATTGGGTCTATCCGAGCGGCTATCGGAGAGAGTATGCTTGAAGGTTCCTCAATGGACCGCATTAAGCGAGTCACAGAAGGACAGTTCa                                       >  1:275351/1‑301 (MQ=255)`  
                                                                                                                                                                                                                                                                         `aCCTAT‑‑‑‑‑‑‑‑‑‑‑‑CGCTATATCTGGCGCTTAGGCTATCAGTGATCAGAACGCTCAGGCCAAAATGATTGCCGCTAAGACCGCTGCTGGTCGTCGTCAAGCTATGGAAATCATGAGGCAGACGAACATCCAGAATGCTGACCTGTCGTTGCAAGCTCGAAGTAAACTTGAGGAAGCGTCCGCCGAGTTGACCCAACAGAACATGCAGAAGGTCCAAGCTATTGGGTATATCCGAGCGGCTATCGGAGAGAGTATGCTTGAAGGTTCCTCAATGGACCGCATTAAGCGAGTCACAGAAGGATAGTTctgg                                     >  1:446530/1‑298 (MQ=255)`  
                                                                                                                                                                                                                                                                         `aCCTAT‑‑‑‑‑‑‑‑‑‑‑‑CGCTATATCTGGCGCTCAGGCTATCAGTGATCAGAACGCTCAGGCCAAAATGATTGCCGCTCAGACCGCTGCTGGTCGTCGTCAAGCTATGGAAATCATGAGGCAGACGAACATCCAGAATGCTGACCTATCGTTGCAAGCTCGAAGTAAACTTGAGGAAGCGTCCGCCGAGTTGACCCCACAGAACATGCAAAAGGTCCAAGCTATTGGGTCTATCCGACCGGCTATCGGAGAGAGTATGCTTGAAGGTTCCTCAATGGACCGCATTAAGCGAGTCACAGAAGGACAGTTCAtt                                     >  1:163622/1‑301 (MQ=255)`  
                                                                                                                                                                                                                                                                           `cTAT‑‑‑‑‑‑‑‑‑‑‑‑CGCTATACCTGGCGCCCAGGCTATCAGTGATCAGAACGCTCAGGCCAAAATGATTGCCGCTCAGACCGCTGCTGGTCGTCGTCAAGCTATGGAAAGCATGAGGCAGACGAACATCCAGAATGCTGACCTATCGTTGCAAGCTCGAAGTAAACTTGAGGAAGCGTCCGCCGAGTTGACCTCACAGAACATGCAGAAGGTCCAAGCTATTGGGTCTATCCGAGCGGCTATCGGAGAGAGTATGCTTGAAGGTTCCTCAATGGACCGCATTAAGCGAGTCACAGAAGGACAGTTCATTCg                                   <  1:57329/301‑1 (MQ=255)`  
                                                                                                                                                                                                                                                                             `aT‑‑‑‑‑‑‑‑‑‑‑‑CGCTATATCTGGCGCTCAGGCTATCAGTGATCAGAACGCTCAGGCCAAAATGATTGCCGCTCAGACCGCTGCTGGTCGTCGTCAAGCTATGGAAATCATGAGGCAGACGAACATCCAGAATGCTGACCTATCGTTGCAAGCTCGAAGTAAACTTGAGGAAGCGTCCGCCGAGTTGACCCCACAGAACATGCAGAAGGTCCAAGCTATTGGGTCTATCCGAGCGGCTATAGGAGAGAGTATGCTTGAAGGTTCCTCAATGGACCGCATTACGCGAGTCACAGAAGGACAGTTCATTCggg                                 >  1:222904/1‑301 (MQ=255)`  
                                                                                                                                                                                                                                                                                           `cGCTATATCTGGCGCTCAGGCTATCAGTGATCAGAACGCTCAGGCCAAACTGATTGCCGCTCAGCCCGCTGCTGGTCGTCGTCAAGCTATGGAAATCATGAGGCAGACGAACATCCAGAATGCTGACCTATCGTTGCAAGCTCGAAGTAAACTTGAGGAAGCGTCCGCCGAGTTGACCTCACAGAACATGCAGAAGGTCCAAGCTATTGGGTCTATCCGAGCGGCTATCGTAGAGATTATGCTTGAAGGTTCCTCAATGGACCGCATTAAGCGAGTCACAGAAGGTCAGTTAAGTAGGGaa                               >  1:249707/1‑301 (MQ=255)`  
                                                                                                                                                                                                                                                                                           `cGCTATATCTGGCGCTCAGGCTATCAGTGATCAGAACGCTCAGGCCAAAATGATTGCCGCTCAGACCGCTGCTGGTCGTCGTCAAGCTATGGAAATCATGAGGCAGACGAACATCCAGAATGCTGACCTATCGTTGCAAGCTCGAAGTAAACTTGAGGAAGCGTCCGCCGAGTTGACCCCACAGAACATGCAGAAGGTCCAAGCTATTGGGTCTATCCGAGCGGCTATCGGAGAGAGTATGCTTGAAGGTTCCTCAATGGACCGCATTAAGCGAGTCACAGAAGGAcctgtctcttataca                               >  1:301414/1‑287 (MQ=255)`  
                                                                                                                                                                                                                                                                                                      `gcgcTCAGGCTATCAGTGATCAGAACGCTCAGGCCAAAATGATTGCCGCTCAGACCGCTGCTGGTCGTCGTCAAGCTATGGAAATCATGAGGCAGACGAACATCCAGAATGCTGACCTATCGTTGCAAGCTCGAAGTAAACTTGAGGAAGCGTCCGCCGAGTTGACCCCACAGAACATGCAGAAGGTCCAAGCTATTGGGTCTATCCGAGCGGCTATCGGAGAGAGTATGCTTGAAGGTTCCTCAATGGACCGCATTAAGCGAGTCACAGAAGGACAGTTCATTCGGGAAGCCAATATGGt                    >  1:38943/1‑301 (MQ=255)`  
                                                                                                                                                                                                                                                                                                           `cAGGCTAGCAGTGATCAGAACGCTCAGGCCAAAATGATTGCCGCTCAGACCGCTGCTGGTCGTCGTCAAGCTATGGAAATCATGAGGCAGACGAACATCCAGAATGCTGACCTATCGTTGCAAGCTCGAAGTAAACTTGAGGAAGCGTCCGCCGAGTTGACCTCACAGAACATGCAGAAGGTCCAAGCTATTGGGTCTATCCGAGCGGCTATCGGAGAGAGTATGCTTGAAGGTTCCTCAATGGACCGCATTAAGCGAGTCACAGAAGGACAGTTCATTCGGGAAGCCAATATGGTAACTg               <  1:380505/301‑1 (MQ=255)`  
                                                                                                                                                                                                                                                                                                             `ggCTATCAGTGATCAGAACGCTCAGTCCAAAATGATTGCCGCTCAGACCGCTGCTGGTCGTCGTCAAGCTATGGAAATCATGAGGCAGACGAACATCCAGAATGCTGACCTATCGTTGCAAGCTCGAAGTAAACTTGAGGAAGCGTCCGCCGAGTTGACCCCACAGAACATGCAGAAGGTCCAAGCTATTGGGTCTATCCGAGCGGCTATCGGAGAGAGTATGCTTGAAGGTTCCTCAATGGACCGCATTAAGCGAGTCACAGAAGGACAGTTCCTTCGGGAAGCCAATATGTTAACTgct             >  1:431580/1‑299 (MQ=255)`  

                                                                                                                                                                                                                                                                                                                        `|`                                                                                                                                                                                                                                                                                                              
`GTGCTGGTTCGTTACGAGCGACCAAGTGTGGCGACTTAGTGGAAAGGCTAAGCGAAAGTTCCGTAAGTTAATCATGGAGTATCGCGATAAGATGCTTGAGAAGTATGATACTCTTTGGAATTACGTATGGGTAGGCAATACGTCCCACATTCGTTTCCTCAAGACTATCGGTGCGGTATTCCATGAAGAGTACACACGAGATGGTCAATTTCAGTTATTTACAATCACGAAAGGAGGATAACCATATGTGTTGGGCAGCCGCAATACCTAT‑‑‑‑‑‑‑‑‑‑‑‑CGCTATATCTGGCGCTCAGGCTATCAGTGGTCAGAACGCTCAGGCCAAAATGATTGCCGCTCAGACCGCTGCTGGTCGTCGTCAAGCTATGGAAATCATGAGGCAGACGAACATCCAGAATGCTGACCTATCGTTGCAAGCTCGAAGTAAACTTGAGGAAGCGTCCGCCGAGTTGACCTCACAGAACATGCAGAAGGTCCAAGCTATTGGGTCTATCCGAGCGGCTATCGGAGAGAGTATGCTTGAAGGTTCCTCAATGGACCGCATTAAGCGAGTCACAGAAGGACAGTTCATTCGGGAAGCCAATATGGTAACTGAGAACTATCGCCG  >  NC_001604.1/27483‑28083`

  

Alignment Legend

Aligned base mismatch/match (shaded by quality score): `ATCG/ATCG` `< 3 ≤` `ATCG/ATCG` `< 10 ≤` `ATCG/ATCG` `< 20 ≤` `ATCG/ATCG` `< 34 ≤` `ATCG/ATCG` `< 38 ≤` `ATCG/ATCG`

Unaligned base: `atcg`    Masked matching base: `atcg`    Alignment gap: `‑`    Deleted base: `‑`
